# Transient elastography in adult patients with cryptic dyskeratosis congenita reveals subclinical liver fibrosis: a retrospective analysis of the Aachen telomere biology disease registry

**DOI:** 10.1186/s13023-021-02024-8

**Published:** 2021-09-26

**Authors:** Mareike Tometten, Martin Kirschner, Susanne Isfort, Marie-Luise Berres, Tim H. Brümmendorf, Fabian Beier

**Affiliations:** 1 Department of Hematology, Oncology, Hemostaseology and Stem Cell Transplantation, Medical Faculty, RWTH Aachen University, Aachen, Germany; 2 Center for Integrated Oncology Aachen Bonn Cologne Duesseldorf (CIO ABCD), Aachen, Germany; 3 Department of Internal Medicine III, Medical Faculty, RWTH Aachen University, Aachen, Germany

**Keywords:** Telomere biology disorder, Dyskeratosis congenital, Transient elastography, Liver

## Abstract

**Background:**

Telomere biology disorders (TBD) such as dyskeratosis congenita (DKC) lead to progressive multi-organ failure as impaired telomere maintenance disturbs cellular proliferative capacity. A wide range of hepatic manifestations from asymptomatic liver enzyme elevation to overt liver fibrosis/cirrhosis can be observed in TBD patients. However, the incidence of hepatic involvement remains unknown. Non-invasive transient elastography (TE) predicts early fibrosis by measuring liver stiffness and may uncover subclinical liver damage in TBD patients.

**Methods:**

Liver screening procedures of nine TBD patients from the Aachen TBD Registry are being presented retrospectively*.* Following clinical suspicion, TBD was diagnosed using flow-FISH with telomere length (TL) below the 1% percentile and confirmed by next-generation sequencing (NGS) detecting pathogenic mutations in telomere maintenance genes TERC or TERT.

**Results:**

In all patients, TBD was first diagnosed in adulthood. Patients showed normal to slightly elevated liver function test parameters. Hepatic ultrasound revealed inhomogeneous parenchyma in seven (77.7%) and increased liver echogenicity in four patients (44.4%). Median liver stiffness was 10.7 kilopascal (kPa) (interquartile range 8.4, 15.7 kPa). Using 7.1 kPa as cut-off, 88.8% of patients were classified as moderate fibrosis to cirrhosis.

**Conclusion:**

Subclinical chronic liver involvement is frequent in patients with adult-onset TBD. TE could have a valuable role in the routine work-up of patients with telomere disorders including DKC for early detection of patients at risk for liver function impairment.

**Supplementary Information:**

The online version contains supplementary material available at 10.1186/s13023-021-02024-8.

## Background

Telomeres represent a unique repetitive DNA structure protecting the chromosomes’ ends. With each cell division, telomere length shortens thereby limiting the proliferative capacity of human somatic cells [1]. Telomeres can be elongated by the enzyme telomerase. Telomere biology disorders (TBD) are a paradigmatic disease to study the systemic consequences of impaired telomere homeostasis. TBDs are characterized by premature shortening of telomeres mostly due to germline mutations in the telomerase complex. Clinically, the most prominent manifestation of TBD is dyskeratosis congenita (DKC). Classical DKC feature is the mucocutaneous triad of oral leukoplakia, nail dysplasia and abnormal skin pigmentation [2]. First manifestation is frequently in childhood or young adulthood with multiorgan involvement including bone marrow failure (BMF), liver fibrosis/cirrhosis and pulmonary fibrosis. In adults, so called “cryptic” variants without typical clinical DKC features are frequently observed mimicking e.g. aplastic anemia or idiopathic lung fibrosis [2, 3]. Importantly, symptomatic affection of additional organs such as liver, gut and others can be found in patients with cryptic variants.

Functional screening for TBD is carried out by measuring the telomere length (TL) in peripheral blood leukocytes (PBL) [4–6]. TBD patients generally have a TL below the 1% percentile of normal controls [7]. Diagnosis is established based on TL, detection of mutations in known genes affecting telomere maintenance, family history and presence of typical DKC symptoms.

Few is known about hepatic involvement in patients with telomere disease, given incidences are ranging from about 10 to 40% [8–10]. Liver abnormalities can vary from asymptomatic elevation of liver enzymes up to cryptogenic liver fibrosis and cirrhosis [8]. Routinely performed liver biopsy may be complicated by the presence of bone marrow failure related thrombocytopenia. Moreover, due to possible complications, the risk–benefit balance of liver histology has been questioned lately, especially on the ground of recent availability of alternative options, consisting in non-invasive imaging [11]. However, early detection of chronic liver disease (CLD) may improve the management of DKC patients, not least because TBD patients can suffer from hepato-pulmonary syndrome, and fatal liver complications after bone marrow transplantation have been described [12, 13]. Moreover, recommendations to limit cofactors for CLD progression such as obesity or alcohol consumption as well as screening for hepatocellular carcinoma would be required in patients with affected livers.

Transient elastography (TE) is a non-invasive method that predicts early liver fibrosis. Vibration generates a mechanical wave speeding across the hepatic parenchyma. The wave´s velocity is converted into measures of liver stiffness (in kilopascal, kPa) and correlates with the severity of fibrosis [11, 14]. Of note, cut-offs for advanced fibrosis differ regarding the underlying liver disease [15–17]. To the best of our knowledge, there are no data investigating the liver in TBD patients by using TE.

Here, we present the results of liver imaging procedures including TE screening in a small cohort of cryptic adult TBD patients included in the *Aachen telomere biology disease registry (ATBDR)*.

## Results

### Patient characteristics

We retrospectively identified nine patients with adult-onset, cryptic TBD and TE/liver ultrasound results available. Detailed patient characteristics are shown in Table 1. Mean age at diagnosis was 38.3 ± 13.4 years. Six patients were males (male/ female ratio 2/1). 44.4% (n = 4) had a positive family history regarding typical TBD features (see Additional file 1: Table S1). Telomere length was below the first percentile in lymphocytes and granulocytes in all patients. Mutations in the TERC gene were found in five patients, TERT mutations in four patients. All patients had cryptic variant of TBD primarily manifesting in young adulthood with a mean age of onset of 30.0 ± 12.7 years. Manifestations of first presentation were cytopenia (n = 6), liver cirrhosis (n = 2) and lung fibrosis (n = 1). One of the cytopenic patients also had elevated liver enzymes. Median time from primary manifestation (PM) to diagnosis was two years (range 0–14 years). Predominantly affected organ systems were bone marrow (100%) and lung (66.6%). Regarding the bone marrow function, 88.9% (n = 8) had thrombocytopenia (common toxicity criteria, CTC grade 2 or more, see Table 2). None of the patients received regular blood transfusions. 66.6% (n = 6) of the patients had a suspected liver manifestation according to the medical history (liver cirrhosis, n = 3; liver fibrosis, n = 1; elevated liver enzymes, n = 1) and one patient had liver biopsy showing nodular regenerative hyperplasia. No patient presented with classical DKC triad or enteropathy. Seven and six patients had a negative hepatitis B and C serology, respectively. No serology data for hepatitis B and C were available in 2 patients and 3 patients, respectively. One patient had a body mass index of 29, all others presented with normal nutritional status. Only one patient had a positive history for alcohol abuse, one patient was a never drinker, all others had alcohol consumption < 12.5 g/die. Additional correlation analysis was not carried out due to the small number of patients.

**Table 1:** Anagraphic and genetic data of the nine analyzed patients

Age, (y) mean ± SD	
At PM	30.0 ± 12.7
At diagnosis	38.3 ± 13.4
At TE	41 ± 12.0
Sex, male, no (%)	6 (66.6%)
Type of DKC (%)	Cryptic variant (100%)
Telomer length < 1st percentile, no (%)	
Lymphocytes	9 (100%)
Granulocytes	9 (100%)
Affected gene mutation, no (%)	
TERC	5 (55.6%)
TERT	4 (44.4%)
Gene variants	
TERC	n.54_57del
	n. 73G > A
	cn.107G > C
	n.128A > G
	n. 73 G > A
TERT	c.2639C > T, [p.A880V]
	c. 3257G > A, [p. R1086H]
	c. 2059G > A, [p. R972H]
	c.2147C > T, [p. A716V]
Additional organ involvement, no (%)	
Bone marrow	9 (100)
Lung	6 (66.6)
Liver^†^	6 (66.6)
Hair	5 (55.6)
Others (nails, heart, endometrium, eye)	1 (12.5) each

*Y* years, *SD* standard deviation, *PM* primary manifestation, *TE* transient elastography, *No* number of patients, *DKC* dyskeratosis congenital, *TERC* telomerase RNA component, *TERT* telomerase reverse transcriptase, *HET* heterozygous

All DKC-confirming mutations were heterozygous; ^†^Liver involvement stated before TE

**Table 2:** Laboratory parameters of the nine analyzed patients

Laboratory parameters, normal values	Median (IQR)	CTC grade
Platelets (/nl), 150–400	38 (16, 67)	3
Marker of hepatocyte damage		
ALT (U/l), ♂ < 50, ♀ < 35	35 (29, 56)	0
AST (U/l), ♂ < 50, ♀ < 35	46 (30, 66)	0 (♂),1 (♀)
Marker of impaired liver synthesis		
INR	1.14 (1.08, 1.25)	0
Albumin (g/dl), 3.5–5.2	4.1 (3.7, 4.5)	0
PT(s), 25.1–36.5	30.1 (29.7, 33.8)	0
Marker of cholangiopathy		
Total Bilirubin (mg/dl), < 1.2	1.13 (0.36, 1.77)	0
ALP (U/l), ♂ 40–130, ♀ 35–105	97 (70, 160)	0

*IQR* interquartile range, *CTC* common toxicity criteria, *ALT* alanine aminotransferase, *AST* aspartate aminotransferase, *ALP* alkaline phosphatase, *INR* international normalized ratio, *PT* prothrombin time

### Biochemical liver pattern

Serum liver enzymes alanine aminotransferase (ALT) and aspartate aminotransferase (AST) as markers of hepatocyte damage were not or only marginally increased above upper limit of normal (ULN) in all patients. Similar, international normalized ratio (INR), albumin and prothrombin time (PT) as marker of impaired liver synthesis did not show relevant abnormalities (CTC grade 0–1), only one patient (11%) had an increased INR (CTC grade 2). Applying markers of cholangiopathy, bilirubin and alkaline phosphatase (ALP), we found, that only two patients (22%) showed increased bilirubin. Platelets were found to be decreased in 8 of 9 patients rather as a manifestation of BMF than as sign of portal hypertension, determined by ultrasound. Based on the parallel existing BMF in all patients, platelets were thought to be not applicable for determining liver status, e. g. as part of the Fib4 score, an indicative parameter for liver fibrosis. Patient´s laboratory parameters are shown in Table 2.

### Liver ultrasound and stiffness

The most common finding identified by hepatic ultrasound was unspecific inhomogenous parenchyma in 77.7% (n = 7) of the patients. 55.6% (n = 5) showed increased liver echogenicity, 33% (n = 3) had a nodular contour. One patient (11%) had cavernous transformation of the portal vein after splenectomy, in further six patients portal vein flow was normal, one patient without data for portal vein flow showed normal size of the spleen. Eight patients had at least one ultrasound liver alteration.

**Fig. 1:**
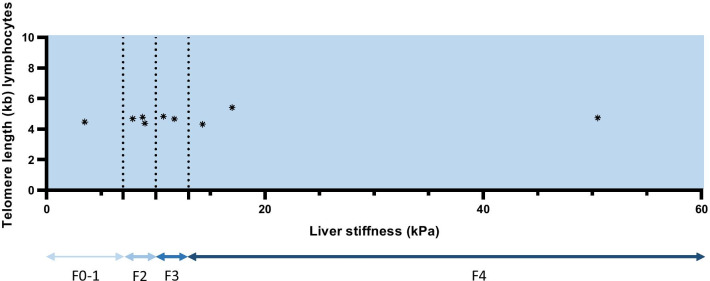
Distribution of liver stiffness in kilopascal (kPa) and fibrosis (F) stage 0–4 in nine telomere biology disorder (TBD) patients. *Marks each individual patient. Eight patients showed liver fibrosis, F ≥ 2

Median fibrosis stage was 10.7 kPa (IQR 8.4, 15.7 kPa). The percentage of liver stiffness ≤ 7 kPa, 7.1–9.9 kPa, ≥ 10 kPa and ≥ 13 kPa was 11.1%, 33.3%, 22.2% and 33.3% respectively. Accordingly, 88.8% of the patients were categorized as fibrosis stage ≥ 2, only one patient was assigned to mild signs of liver fibrosis (see Table 3; Fig. 1). We found two patients without previously suspected liver manifestation who had fibrosis stage ≥ 2 (8.8 kPa and 17 kPa, respectively). Of those, one patient showed slightly increased echogenicity, one had normal liver ultrasound and showed fibrosis stage 4. All patients with previously suspected liver manifestation had liver fibrosis stage ≥ 2.

**Table 3:** Liver ultrasound and transient elastography results of the nine analyzed patients

	No (%)
Hepatic ultrasound	
Inhomogenous parenchyma	7 (77.7)
Increased echogenicity	5 (55.6)
Nodular contour	3 (33.3)
Evidence of portal hypertension	1 (11.1)
Fibrosis stage by transient elastography	
0/1	1 (11.1)
2	3 (33.3)
3	2 (22.2)
4	3 (33.3)

*No* number of patients

## Discussion

Adequate monitoring of patients with TBD is important for uncovering organ manifestations to be implemented into patient care, prevent unnecessary toxicities and potentially to trigger novel treatment strategies [18]. Beyond bone marrow and lung, the liver is the third important major organ affected by TBD [3]. Disadvantageously, liver fibrosis and cirrhosis are asymptomatic until complications of chronic liver injury occur [19].

Regarding the role of telomeres in chronic liver injury, it is hypothesized that telomere shortening results in replicative senescence or apoptosis of hepatocytes and finally in liver cirrhosis [20]. In patients with sporadic liver cirrhosis, mutations of the telomerase complex were found with increased allele frequency of 0.017 compared to 0.003 in non-cirrhotic patients, further substantiating a critical role of telomeres in the pathogenesis of liver cirrhosis [21]. Therefore, screening for telomere disease in case of cryptogenic liver fibrosis and/ or cirrhosis is recommended.

The liver has excessive proliferative capacity. Unimpaired telomere maintenance has shown to be crucially important for liver regeneration [22]. However, few is known about hepatic manifestations in DKC and TBD patients, respectively, not least since telomerase mutations are risk factors for liver impairment, but also individual environmental factors contribute to the development of liver disease [8]. A recent work of Kapuria et al. showed increased liver echogenicity by ultrasound in approximately 40% of the TBD patients. However, identification of hepatic involvement was carried out using suggestive clinical features as elevated liver enzymes were found in 98% of the identified patients. Patients with milder forms of hepatic affections were thus not included in recent studies leading to a major systemic bias.

In our study, we systematically analyzed patients of the ATBDR with proven TBD for liver manifestations and found variable clinical presentations. Eight of the nine adult patients with telomere disease in our cohort displayed some signs of unspecific imaging abnormalities as determined by liver ultrasound. No patient showed a significant elevation of liver enzymes, only two patients had an elevation of bilirubin CTC grade ≥ 2.

Liver biopsy is the gold standard to accurately stage and grade chronic liver diseases [23]. To clearly determine fibrosis stages in patients with TBD, histological investigation would be necessary. However, due to mild forms of hepatic affection and the increased risk of bleeding caused by the concomitant BMF, only few patients receive a diagnostic liver biopsy, and sequential screening is not convenient [10]. This was applicable to 8 of 9 patients in our cohort presenting with thrombocytopenia CTC ≥ 2. Lacking systematic studies, TBD can display a variety of histological features like nonalcoholic fatty liver disease, inflammation, early fibrosis to cirrhosis, nodular regenerative hyperplasia and iron accumulation even in absence of history of blood transfusion [8, 10, 24]. In our cohort, only one patient had a history of liver biopsy, which showed steatosis with minimal inflammation but no signs of cirrhosis.

Along the same line, accurate knowledge about the liver status is of utmost importance for the proper management of patients with telomere disease after bone marrow transplantation for BMF. There is a substantial risk of toxicity in the transplant setting leading to a poor long-term survival of patients with DKC after allo-transplantation. For instance, Rocha et al. reported about five patients with DKC, who experienced unusual complications after bone marrow transplantation, amongst others especially liver failure [13]. Based on their data, they urgently recommended a complete evaluation of the liver beside respiratory tract and endothelial parameters before transplant. Transplantation could be initiated at an earlier point of time before liver damage progresses. Moreover, in the transplant setting modified protocols are recommended because of the risk of pulmonary and liver fibrosis in these patients [25, 26]. Also, the occurrence of severe hepato-pulmonary syndrome in TBD patients has been described [24].

Implementation of non-invasive methods for the assessment of chronic liver diseases represents an elegant method to overcome the problems accompanying liver biopsy. TE represents a useful tool to identify and stage liver fibrosis and is validated for different liver diseases such as non-alcoholic fatty liver disease, chronic viral hepatitis or autoimmune hepatitis [23]. Cut-offs depend upon the etiology of the underlying liver disease, complicating the recommendation of specific cut-offs, and no consensus has been reached until now [15, 23]. However, TE is broadly available, has a good reproducibility and diagnostic accuracy for staging liver fibrosis. It is a cost-effective diagnostic tool, and sequential testing is easy to implement. Limitations of TE are due to the analysis of only a small volume of the liver at one time and the operator-dependency [27] and that analysis was not carried out blinded. Excessive iron deposit due to repeated blood transfusions can also contribute to TE alterations [28]. However, no relevant transfusion history was reported for our patients.

Our study extends the non-invasive management for patients with telomere disease. We show, that unexpectedly, eight of nine patients—even in the absence of significant liver parameter abnormalities—had liver fibrosis stage 2 or higher. Moreover, two of the three patients, where no liver manifestation was suspected in the past medical history exhibited liver fibrosis. Using TE, we were able to demonstrate that adult TBD patients frequently exhibit subclinical hepatic manifestations.

## Conclusions

We describe here for the first time the implementation of non-invasive TE in the management of patients with telomere disorders. We confirm, that subclinical chronic liver disease is frequent in patients with adult-onset, cryptic TBD. Recognizing liver damage draws the consequence of avoiding noxious agents, screening for hepatocellular carcinoma, earlier transplantation and a special peri-transplantation setting. TE could have a valuable role in determining liver involvement in patients even in absence of abnormal laboratory parameters. However, prospective data are need to recommend the implementation of TE in the routine work-up in order to detect subclinical liver fibrosis/cirrhosis and improve management in this highly vulnerable group of patients.

## Methods

### Patients

Retrospective analysis comprised patients from the ATBDR with confirmed diagnosis of TBD and results of TE/liver ultrasound results available. There were no exclusion criteria. In general, patients are included in the ATBDR when the treating physician suspects DKC or telomeropathy on a clinical basis and/or according to the recommendations of the German Society of Hematology and Oncology (DGHO) for the diagnostic workup of aplastic anemia published via Onkopedia (www.onkopedia.de). After written informed consent is obtained, demographic and clinical data are collected. Peripheral blood (PB) samples are taken according to the approval by the local ethics committee (EK206/09, 5 January 2010, RWTH Aachen University). Samples were analyzed by flow-FISH, and, if deemed critically short, further analyzed using Next-generation sequencing (NGS) to screen for disease causing mutations. Patients were eligible if they had liver ultrasound and TE as well as a confirmed genetic mutation in a DKC related gene. Unblinded liver ultrasound and TE were carried out following standard procedures and were taken regardless of presence or suspicion of liver impairment. Cut-offs of liver stiffness were used as proposed lately [15]. Of note, there are no recognized cut-offs for Telomere Biology Disorders. Therefore, we chose cut-offs in analogy to the proposed algorithm of Friedrich-Rust et al. [15]: ≤ 7 kPa minimal or no presumed fibrosis (stage 0/1), 7.1–9.9 kPa presumed moderate fibrosis (stage 2), ≥ 10 kPa presumed severe fibrosis (stage 3) and ≥ 13 kPa presumed cirrhosis (stage 4). Radiographic and laboratory data were obtained reviewing the computerized medical records.

### Flow-FISH

For TL measurement, flow-FISH was used according to previously described protocols, and TL is indicated in kilobases (kb) [29–31].

### Targeted amplicon sequencing

NGS (MiSeq®, Illumina, Germany) was done as previously described [32]. Library preparation was done using the TruSeqRCustom Amplicon kit (Illumina). Genetic variants/ heterozygous mutations in telomere maintenance genes were screened using a self-designed panel containing the entire coding sequences for CTC1, DKC1, NHP2, NOP10, RTEL1, TERC, TERT, TCAB1, USB1, and exon 6 of TINF2 [32].

### Statistical analysis

Results are expressed by mean ± standard deviation (SD) and median with interquartile range (IQR). Analysis was done using GraphPad Prism (GraphPad Software version 9.0.0, La Jolla, CA, USA).

## Supplementary Information

**Additional file 1. Table S1**: Patient specific clinical manifestations and family history.

## Data Availability

Additional data and materials can be provided upon request to the corresponding author.
