# Supplementary material for: Transient elastography in adult patients with cryptic dyskeratosis congenita reveals subclinical liver fibrosis: a retrospective analysis of the Aachen telomere biology disease registry
Source: Orphanet J Rare Dis. 2021 Sep 26;16:395. doi: 10.1186/s13023-021-02024-8 (PMC8474920; doi:10.1186/s13023-021-02024-8)
Supplement: Supplementary file 1 — Additional file 1. Table S1: Patient specific clinical manifestations and family history. [file 13023_2021_2024_MOESM1_ESM.docx]

**Suppl. Table 1: Patient specific clinical manifestations and family history**

| UPN | Genotype | Additional clinical manifestations | Family history |
| --- | --- | --- | --- |
| 1  2  3  4  5  6  7  8  9 | **TERC**  **TERT**  **TERT**  **TERC**  **TERT**  **TERC**  **TERC**  **TERC**  **TERT** | Early hair greying, BMF, ILD, endometrial cancer  Early hair greying, BMF, ILD  BMF, ILD  BMF  Early hair greying, BMF, ILD, eye  BMF, nail dystrophy  BMF, ILD, nail dystrophy  Early hair greying, BMF, cardiomyopathy  Early hair greying, BMF, ILD | Sibling with BMF, father with ILD  Sibling with liver disease and ILD  Mother with BMF  None  None  None  None  Two Sibling with ILD  None |

UPN: Unique patient number, BMF: bone marrow failure, ILD: interstitial lung disease
